# Cortical network characteristics in post-stroke anxiety: an fNIRS-based study

**DOI:** 10.3389/fnins.2025.1708752

**Published:** 2026-01-16

**Authors:** Xue Qian, Qinglei Wang, Jie Wang, Ling Yang, Ayan Geng, Wenjie Xu, Gengjuan Dong, Tongbo Lu, Chuan Guo

**Affiliations:** 1 Changzhou Dean Hospital, Changzhou, Jiangsu, China; 2 Changzhou Mental Health Center, Changzhou, Jiangsu, China; 3 The First Affiliated Hospital with Nanjing Medical University, Nanjing, Jiangsu, China

**Keywords:** cortical network characteristics, functional connectivity, functional near-infrared spectroscopy, post-stroke anxiety, prefrontal cortex, verbal fluency task

## Abstract

**Objective:**

To examine prefrontal hemodynamic changes in patients with post-stroke anxiety (PSA), both at rest and during cognitive task engagement, with the aim of elucidating the underlying neural mechanisms of PSA and identifying potential neural correlates for clinical application.

**Methods:**

Fifty patients with PSA and 45 post-stroke patients without anxiety symptoms were recruited. PSA was diagnosed using the Hamilton Anxiety Rating Scale (HAMA ≥ 7), and comorbid depression was screened using the 17-item Hamilton Depression Rating Scale (HAMD-17 ≥ 8). Patients with significant cognitive impairment were excluded. Functional near-infrared spectroscopy (fNIRS) was used to measure resting-state functional connectivity in the frontopolar cortex (FPC) and dorsolateral prefrontal cortex (DLPFC), as well as task-evoked activation during the verbal fluency task (VFT). Demographic and clinical characteristics showed no significant differences between groups except for stroke type. Between-group comparisons were conducted to identify PSA-related differences in prefrontal network characteristics. Subgroup analyses were performed to explore the influence of comorbid depression on neural alterations.

**Results:**

There were no significant differences between the PSA and non-PSA groups in demographic or clinical characteristics, including age, sex, and disease duration (*P* > 0.05). Compared to the non-PSA group, patients with PSA exhibited significantly reduced activation in the bilateral FPC during the VFT (*P* < 0.05). Within the PSA group, those with comorbid depression showed further reductions in activation in the bilateral FPC and the left DLPFC (*P* < 0.05). No significant differences in resting-state functional connectivity were observed between groups (*P* > 0.05).

**Conclusion:**

Reduced activation in the bilateral FPC may represent a key neural substrate associated with post-stroke anxiety. In addition, altered activation patterns in the bilateral FPC and left DLPFC may reflect neural correlates related to depressive symptoms in patients with PSA, providing candidate targets for future mechanistic and clinical studies.

## 1 Introduction

Stroke remains one of the foremost contributors to fatalities and impairments around the world. Survivors of stroke often face a dual burden, encompassing both physical dysfunction and psychological disorders (Abdelhameed et al., 2024). Post-stroke anxiety (PSA) is the most widespread psychological complication, with prevalence rates ranging from 29.3% to 36.7%, varying at different post-stroke stages (Rafsten et al., 2018; Xiao et al., 2020). PSA not only obstructs neurological recovery but also significantly exacerbates the risk of subsequent post-stroke depression (PSD) and cognitive deficits (Chun et al., 2018; Li et al., 2021). However, the diagnosis and objective evaluation of PSA remain challenging, due to methodological disparities in assessment approaches and the confounding effects of post-stroke aphasia and cognitive deficits (Zhou et al., 2023). Moreover, the neurophysiological mechanisms underlying PSA remain incompletely understood. Therefore, identifying reliable biomarkers for PSA could be pivotal in enhancing both the rehabilitation and prognostic assessment of stroke survivors.

Current research into the neurophysiological mechanisms of PSA predominantly focuses on abnormalities in brain network functions, neurotransmitter imbalances, neuroinflammatory processes, and alterations in neuroplasticity (Chun et al., 2018; Petrovic-Djergovic et al., 2016; Sergeeva et al., 2020). Functional magnetic resonance imaging (fMRI) enables the examination of PSA-related alterations in brain networks by measuring brain metabolic activity. Several studies have shown that the location of the stroke lesion is closely linked to the risk of developing PSA. For instance, Tang et al. (2012) demonstrated through fMRI that infarcts in the right frontal lobe constitute an independent risk factor for PSA. Longitudinal investigations further corroborate that extensive damage to the neural fiber connections within the cerebral hemisphere, occurring within 3 months post-stroke, significantly increases the likelihood of PSA (Li et al., 2019). Furthermore, resting-state fMRI studies have uncovered distinctive brain network characteristics in patients with PSA. Fornito et al. (2015) reported abnormal enhancement of functional connectivity within the cerebellum, brainstem, and right middle frontal gyrus in PSA patients, implicating brain regions involved in emotional regulation, as seen in the prefrontal cortex (PFC), as key contributors to the onset of PSA. Further investigations have emphasized the pivotal role of functional disruption within the default mode network in PSA, with some suggesting that its influence may outweigh that of the lesion site itself (Vicentini et al., 2017). Notably, the heterogeneity in these findings may arise from differences in study designs. fMRI studies have also demonstrated that brain network connectivity is more widespread and stable during task states than resting states. Nevertheless, the application of fMRI in PSA biomarker research and studies examining individual differences remains constrained by methodological limitations, including experimental paradigms and stimulus presentation techniques, which impact both the reliability and clinical applicability of the findings (Peng et al., 2023; Elliott et al., 2020).

Functional near-infrared spectroscopy (fNIRS) is a non-invasive technique for neuroimaging that offers several advantages, such as ease of use and resistance to motion artifacts, enabling the monitoring of hemodynamic activity within the cerebral cortex (Lai et al., 2017; Kumar et al., 2017). In comparison to fMRI, fNIRS boasts superior temporal resolution and is less susceptible to environmental noise and head movement, rendering it particularly well-suited for a range of task scenarios, particularly in patients with functional impairments (Pinti et al., 2020; Balada et al., 2024; Zhuang et al., 2022). As a result, fNIRS offers a viable alternative to fMRI, serving as a reliable approach for studying the brain mechanisms associated with PSA (Yang et al., 2023).

The verbal fluency task (VFT), a commonly employed framework in fNIRS research, can assess the hemodynamic response in the cortical regions during cognitive tasks (Husain et al., 2023). Evidence indicates that VFT activates the PFC, particularly the bilateral dorsolateral prefrontal cortex (DLPFC) (Ho et al., 2020). Many studies have reported that individuals with anxiety exhibit significantly lower activation in the PFC during the VFT. And the activation intensity in the left PFC was negatively correlated with the severity of anxiety symptoms (Wang et al., 2024, 2022; Uchida and Hirao, 2020). In research involving comorbid populations, Hu et al. (2021) employed fNIRS to observe patients with anxiety, depression, and comorbid anxiety-depression during VFT, revealing that all three groups exhibited markedly diminished activation in the left DLPFC. These findings suggest a potential shared neuropathological mechanism, supporting further investigation into their common neurophysiological basis. Furthermore, studies about task-state fNIRS have been utilized to explore neurophysiological distinctions between emotional disorders with overlapping symptomatology. Wen et al. (2021) identified significant fluctuations in PFC hemoglobin concentrations in both depression and anxiety-depression patients during VFT, while resting-state data proved insufficient for distinguishing these groups. Additional research has highlighted that anxiety-depressed patients exhibit significantly elevated activation in the right PFC, compared to non-anxiety-depressed individuals, suggesting that this region may serve as a key biomarker for distinguishing anxiety-depression (Wu et al., 2024). These results emphasize the critical role of task-state research in clarifying the involvement of the PFC in emotional regulation.

From a clinical perspective, clarifying the neural mechanisms underlying post-stroke anxiety is essential for improving its clinical management. A better understanding of PSA-related brain alterations may help clinicians identify patients who are more vulnerable to persistent anxiety symptoms, facilitate early psychological monitoring, and support more individualized rehabilitation planning.

Current fNIRS studies on PSA have primarily focused on overall cortical changes within the PFC, with little attention to the specific cortical networks of its subregions, such as the frontopolar cortex (FPC) and DLPFC. Notably, previous studies have indicated that these regions play central roles in cognitive control, executive function, and emotion regulation, and are closely associated with anxiety and related affective disorders (Koechlin and Hyafil, 2007; White et al., 2023), suggesting that the cortical network features underlying PSA remain insufficiently understood. Alterations in the cortical networks of the FPC and DLPFC may therefore contribute to PSA-related emotional deficits. To address this gap, the present study focuses on four key PFC subregions-the left and right FPC (L-FPC, R-FPC) and the left and right DLPFC (L-DLPFC, R-DLPFC)-using fNIRS to monitor functional connectivity in the PFC during the resting state and cortical activation during the VFT, with the aim of providing new objective evidence for understanding the potential pathogenesis of PSA and improving its clinical diagnosis.

## 2 Materials and methods

### 2.1 Participants

No a priori sample size calculation was performed, as this study was designed as an exploratory investigation, and the sample size was determined by clinical feasibility and recruitment availability during the study period, similar to previous fNIRS studies conducted in stroke populations (Koyanagi et al., 2021).

From December 2024 to March 2025, stroke patients were recruited from Changzhou Dean Hospital, Jiangsu Province, China. Participants were divided into two groups according to their scores on the Hamilton Anxiety Rating Scale (HAMA): the anxiety group (HAMA score ≥ 7) and the non-anxiety group (HAMA score < 7).

The criteria for inclusion were as follows: (1) diagnosis (Chinese Classification of Cerebrovascular Diseases, 2015 revision) of stroke, and, confirmed by CT or fMRI as either cerebral hemorrhage or cerebral infarction; (2) age between 18 and 80 years old; (3) disease duration ranging from 1 to 12 months; (4) no prior use of psychiatric medications or participation in physical rehabilitation; and (5) the ability to complete all tests and provide informed consent.

The criteria for exclusion were as follows: (1) presence of severe speech impairment; (2) Significant cognitive impairment, affect the understanding or task execution; (3) cranial defects or a history of cranioplasty with alloplastic materials; and (4) lesions involving cortical structures.

Initially, 50 patients with PSA and 50 patients without PSA were enrolled. Among the non-PSA group, 3 participants withdrew voluntarily, and 2 were unable to complete the experiment. Consequently, 50 PSA patients and 45 non-PSA patients were considered in the final assessment. Written informed consent was obtained from all participants prior to their enrollment in the study. The study received approval from the Ethics Committee of Changzhou Dean Hospital (CZDALL-2024-004) and registered with the Chinese Clinical Trial Registry (ChiCTR2400092342).

### 2.2 Clinical assessment

All participants were native Mandarin speakers and their right-handedness was verified using the Edinburgh Handedness Inventory. Clinical assessments were performed by a senior psychiatrist who was blinded to the group assignments. Anxiety and depressive symptoms were assessed, respectively using the HAMA and the 17-item Hamilton Depression Rating Scale (HAMD-17).

Because stroke severity and functional independence status may influence emotional symptoms, we additionally assessed participants using the National Institutes of Health Stroke Scale (NIHSS) and the Activities of Daily Living (ADL) scale. These measures were collected to characterize baseline neurological and functional status and to reduce the potential confounding effects of disparities in stroke severity and daily living ability on emotional outcomes.

### 2.3 fNIRS measurement

**Figure 1:**
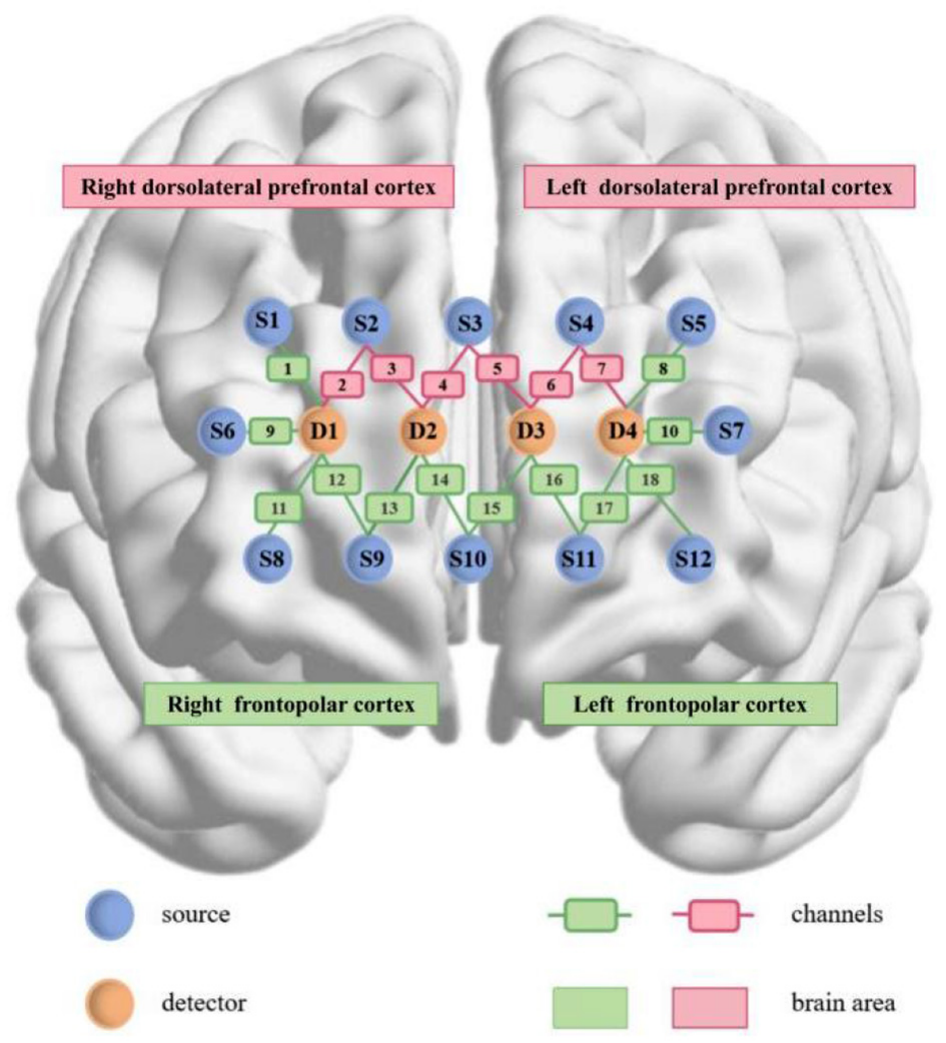
fNIRS Channels and ROI localization map.

Neuroimaging data were collected using fNIRS (NirSmirt-3000A, Danyang Huichuang, China). The system comprises 12 light sources and 4 detectors, resulting in 18 measurement channels. The system measures relative variations in oxygenated hemoglobin (HbO), deoxygenated hemoglobin (HbR), and total hemoglobin (HbT) by transmitting near-infrared light at wavelengths of 730 nm and 850 nm. The source-detector separation was 3 cm, with data sampled at 11 Hz. Optode placement adhered to the international 10–20 EEG system. The following regions of interest (ROIs) were defined: L-FPC (channels 8, 10, 15, 16, 17, 18), R-FPC (channels 1, 9, 11, 12, 13, 14), L-DLPFC (channels 5, 6, 7), and R- DLPFC (channels 2, 3, 4) (see Figure 1).

### 2.4 Experimental design

#### 2.4.1 Resting-state data acquisition

Participants were seated comfortably, with their heads stabilized using a neck support. After the fNIRS cap was fitted, they remained awake with their eyes open in a natural state, avoiding any limb or head movements. Resting-state functional brain data were continuously recorded for a total duration of 6 min.

#### 2.4.2 VFT task data acquisition

**Figure 2:**
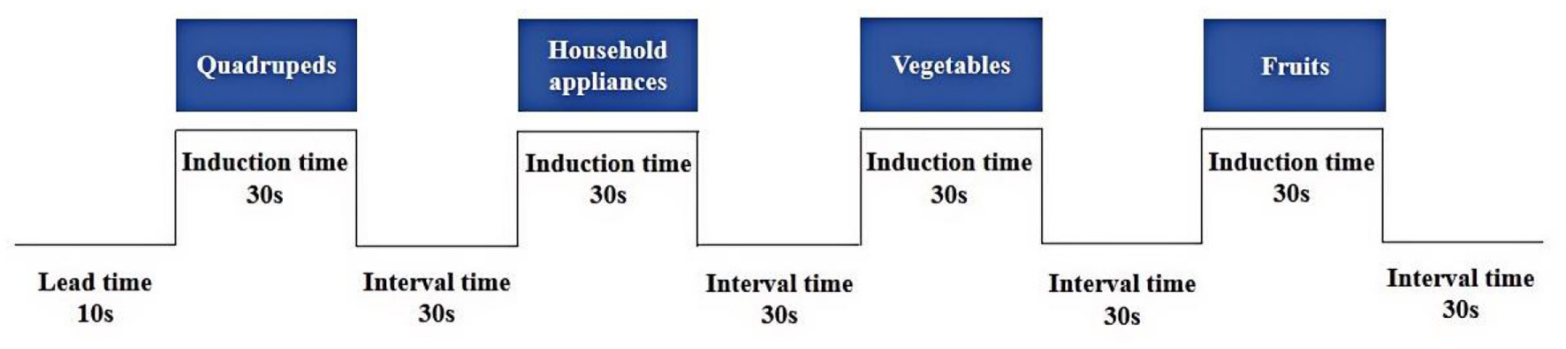
VFT paradigm design.

A standardized block design paradigm was employed, consisting of an initial 10-s baseline followed by four task blocks. Each block included a 30-s activation phase, alternated with a 30-s rest phase. Participants remained seated and were instructed to minimize physical movement during the task. They were instructed to verbally produce as many words as they could within a specified time frame, drawing from predetermined semantic categories (e.g., vegetables, quadrupeds, household appliances, and fruits). The task protocol is depicted in Figure 2. Prior to the task, a brief clinical screening was performed to ensure that participants were alert and able to follow instructions; individuals unable to complete the practice trials were excluded.

### 2.5 fNIRS data processing

Data preprocessing was performed using the Homer2 toolbox of Matlab 2013b software. Raw optical intensity data were transformed into optical density measurements during preprocessing. Motion artifacts caused by head movements were identified using artifact reduction algorithms and corrected via spline interpolation. To eliminate physiological noise and low-frequency drift, a band-pass filter (0.01–0.1 Hz) was applied. The filtered data were subsequently transformed into HbO concentrations based on the modified Beer-Lambert law.

For resting-state analysis, the 1–6 min window post-task onset was used for functional connectivity (FC) analysis. Pearson correlation coefficients (r values) were calculated for the HbO time series between channels, followed by Fisher's r-to-z transformation to normalize the distribution. The resulting z-values represented FC strength between ROIs for statistical analysis.

For task-state analysis, block averaging was applied to the HbO signals from each task trial to mitigate baseline variation. The average ΔHbO during the 0–30 s activation period was calculated for each channel as a measure of activation. Then channel-level values were averaged within each predefined brain region to derive ROI-level activation metrics for statistical analysis.

### 2.6 Statistical analysis

Statistical analyses were performed using SPSS version 26.0. The Shapiro-Wilk test was used to assess data normality. Continuous variables with a normal distribution are expressed as mean ± standard deviation, while non-normally distributed variables are presented as median and interquartile range [M (P25, P75)]. For normally distributed data, independent sample *t*-tests were used for between-group comparisons, whereas the Mann-Whitney U test was applied for non-normally distributed data. Categorical variables were assessed through chi-square tests. Given that lesions in the left and right hemispheres may influence cerebral blood flow, a two-way ANOVA was performed on the fNIRS data, with group and lesion side as independent variables, to examine their effects on changes in brain activation or functional connectivity strength. A *p-value* below 0.05 was deemed statistically significant.

## 3 Results

### 3.1 Demographic and clinical characteristics

Between-group comparisons of demographic and clinical characteristics (Table 1) were performed using independent-samples *t*-tests or Mann-Whitney U-tests for continuous variables, and χ^2^ tests for categorical variables, as appropriate. As shown in Table 1, there were no significant differences between the PSA and non-PSA groups in age, sex, years of education, disease duration, or lesion side (all *P* > 0.05), whereas stroke type differed significantly between groups (*P* = 0.026).

**Table 1:** Demographic characteristics of PSA patients and non-PSA patients.

**Variables**	**PSA group (*N* = 50)**	**Non-PSA group (*N* = 45)**	**t/χ^2^/Z**	***P***
Age (years)	63.66 ± 10.61	59.36 ± 13.13	1.765	0.081
Gender (male/female)	37/13	34/11	0.030	0.862
Years of education (years)	9.00 (9.00, 12.00)	9.00 (9.00, 12.00)	−0.332	0.740
Disease duration (months)	2.00 (1.00, 7.50)	2.00 (1.00, 4.00)	−0.539	0.590
Lesion side (left/right)	19/31	20/25	0.406	0.524
Stroke type (ischemic/hemorrhage)	43/7	30/15	4.975	0.026

Values are presented as mean ± SD for normally distributed variables and median (IQR) for non-normally distributed variables.

Between-group comparisons of clinical scale scores (Table 2) were performed using Mann-Whitney U-tests. As shown in Table 2, the PSA group showed significantly higher HAMA and HAMD-17 scores than the non-PSA group (both *P* < 0.001), whereas no significant between-group differences were observed in ADL and NIHSS scores (all *P* > 0.05).

**Table 2:** Clinical characteristics of PSA patients and non-PSA patients.

**Variables**	**PSA group (*N* = 50)**	**Non-PSA group (*N* = 45)**	***Z***	***P***
HAMA	11.50 (8.00, 15.00)	2.00 (0.00, 3.00)	−8.393	< 0.001
HAMD-17	9.00 (6.75, 11.00)	0.00 (0.00, 3.00)	−7.803	< 0.001
ADL	60.00 (38.75, 71.25)	60.00 (40.00, 85.00)	−0.553	0.580
NIHSS	3.00 (2.00, 7.00)	3.00 (1.00, 5.00)	−1.704	0.099

Values are presented as median (IQR). HAMA, Hamilton Anxiety Rating Scale; HAMD-17, 17-item Hamilton Depression Rating Scale; ADL, Activities of Daily Living scale; NIHSS, National Institutes of Health Stroke Scale.

### 3.2 Hemodynamic responses during the VFT in PSA and non-PSA patients

**Figure 3:**
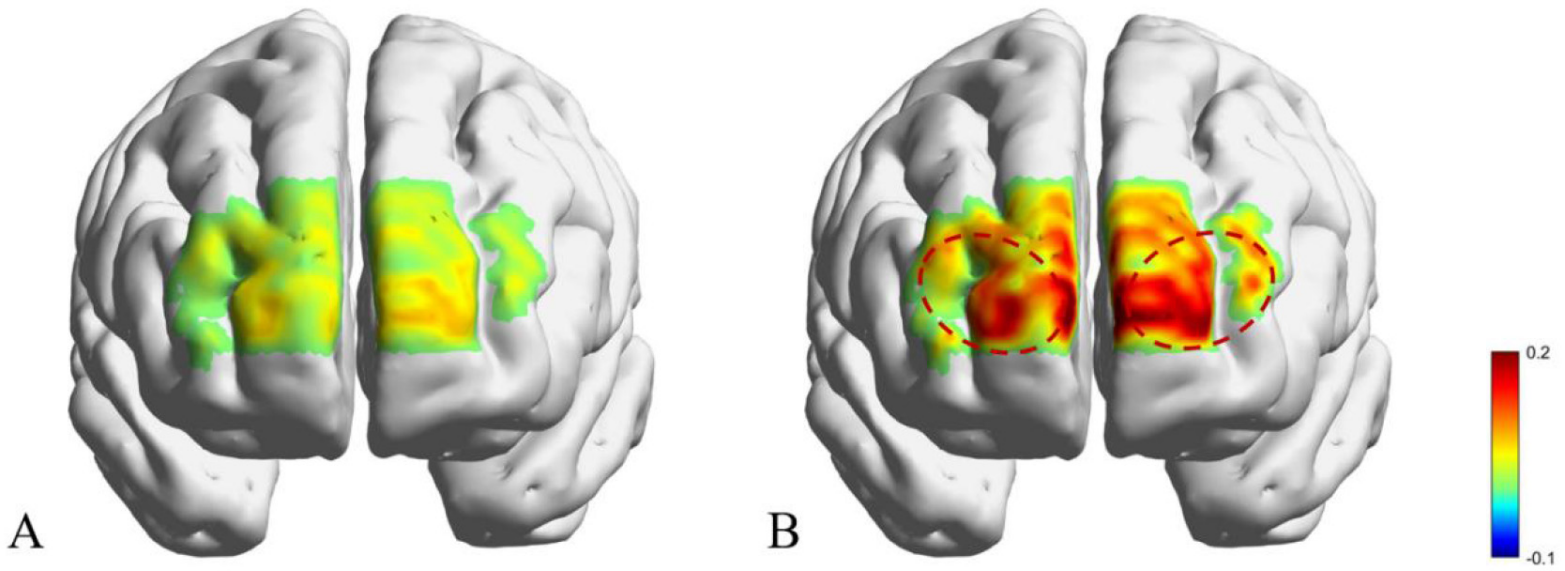
fNIRS-derived cortical activation maps of PSA and non-PSA patients during the VFT. **(A)** Anxiety group; **(B)** Non-anxiety group.

Between-group differences in VFT-related prefrontal activation (Table 3) were examined using ANOVA. As shown in Table 3 and Figure 3, the PSA group exhibited significantly reduced activation in the bilateral FPC during the VFT compared with the non-PSA group (*P* < 0.05), whereas no significant between-group differences were observed in bilateral DLPFC activation (*P* > 0.05). Group and lesion side effects on VFT-related prefrontal activation (Table 4) were examined using two-way ANOVA. No significant main effects of lesion side or group × lesion side interaction effects were observed (all *P* > 0.05).

**Table 3:** Brain activation during VFT with and without anxiety.

**Variables**	**Anxiety group**		**Non-anxiety group**	
	**Left lesion (*****N*** = **19)**	**Right lesion (*****N*** = **31)**	**Left lesion (*****N*** = **20)**	**Right lesion (*****N*** = **25)**
R-FPC	0.044 ± 0.177	0.063 ± 0.146	0.195 ± 0.235	0.108 ± 0.148
R-DLPFC	0.051 ± 0.111	0.035 ± 0.094	0.119 ± 0.188	0.063 ± 0.152
L-DLPFC	0.069 ± 0.150	0.029 ± 0.116	0.138 ± 0.241	0.052 ± 0.158
L-FPC	0.074 ± 0.176	0.066 ± 0.164	0.231 ± 0.250	0.139 ± 0.159

Values are presented as mean ± SD. R-FPC, right frontopolar cortex; L-FPC, left frontopolar cortex; R-DLPFC, right dorsolateral prefrontal cortex; L-DLPFC, left dorsolateral prefrontal cortex. Left lesion and right lesion refer to the hemispheric side of the stroke lesion.

**Table 4:** Comparative analysis of brain activation during VFT with and without anxiety.

**Variables**	**Group effect**		**Lesion side effect**		**Interaction effect**	
	***F***	***P***	***F***	***P***	***F***	***P***
R-FPC	7.191	0.009	0.877	0.351	2.056	0.155
R-DLPFC	2.732	0.102	1.567	0.214	0.490	0.486
L-DLPFC	1.770	0.187	3.280	0.073	0.435	0.511
L-FPC	8.645	0.004	1.626	0.205	1.150	0.286

Values represent F statistics and corresponding P-values from two-way ANOVA (Group × Lesion Side). R-FPC, right frontopolar cortex; L-FPC, left frontopolar cortex; R-DLPFC, right dorsolateral prefrontal cortex; L-DLPFC, left dorsolateral prefrontal cortex.

### 3.3 Resting-state prefrontal functional connectivity in PSA and non-PSA patients

**Figure 4:**
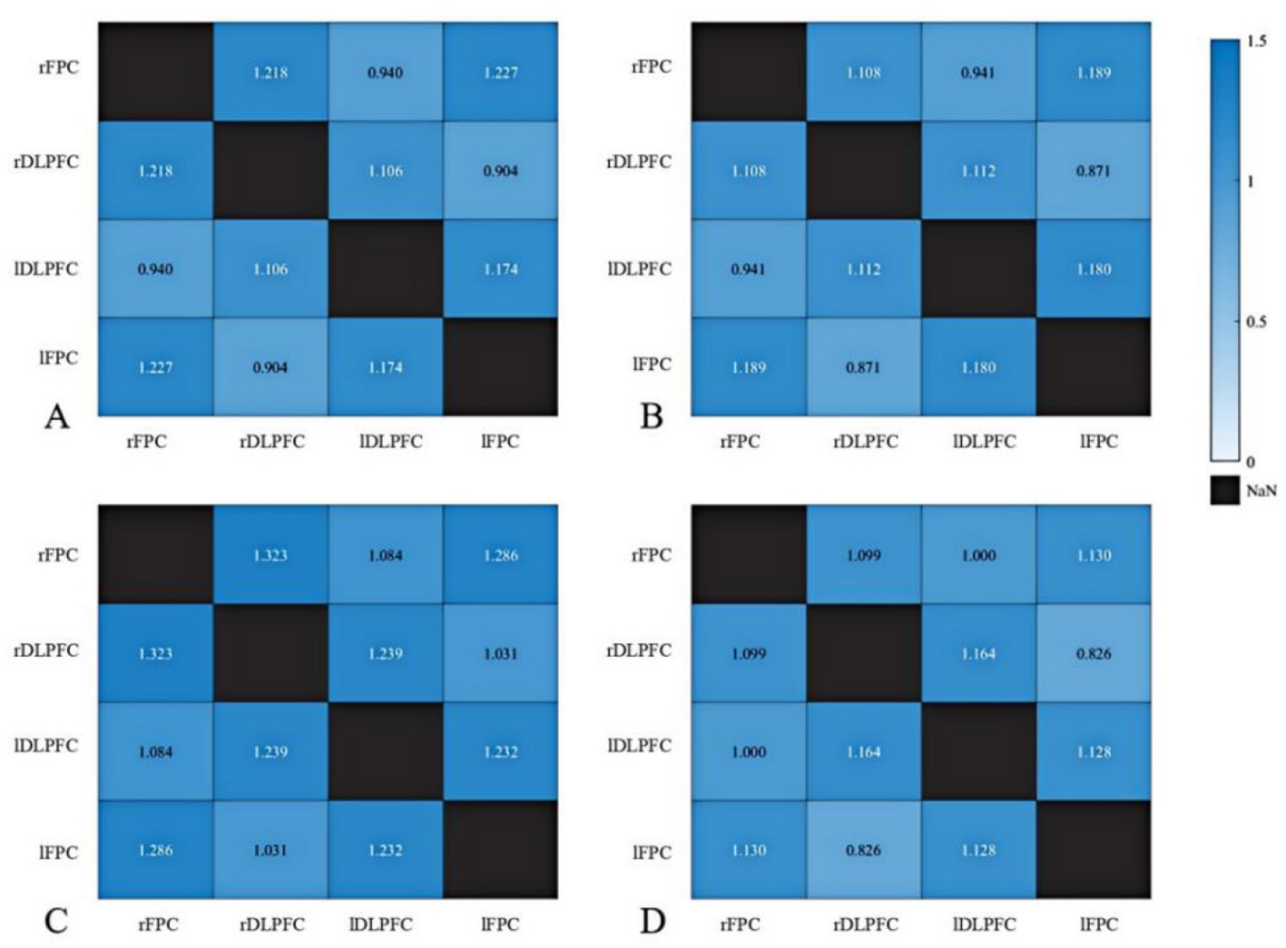
Resting-state brain functional connectivity (z-values) in individuals with and without anxiety. **(A)** Anxiety group with left hemisphere injury; **(B)** Anxiety group with right hemisphere injury; **(C)** Non-anxiety group with left hemisphere injury; **(D)** Non-anxiety group with right hemisphere injury.

Group and lesion side effects on resting-state prefrontal functional connectivity (Table 5) were examined using two-way ANOVA. No significant main effects of group, lesion side, or group × lesion side interaction effects were observed (all *P* > 0.05; Table 5 and Figure 4).

**Table 5:** Comparison of resting-state brain functional connectivity with and without anxiety.

**Variables**	**Group effect**		**Lesion side effect**		**Interaction effect**	
	***F***	***P***	***F***	***P***	***F***	***P***
R-FPC-R-DLPFC	0.176	0.675	2.149	0.146	0.248	0.620
R-FPC- L-DLPFC	1.085	0.300	0.182	0.671	0.183	0.670
R-FPC-L-FPC	0.000	1.000	1.205	0.275	0.441	0.508
R-DLPFC -L-DLPFC	0.769	0.383	0.109	0.742	0.148	0.702
R-DLPFC-L-FPC	0.163	0.687	1.323	0.253	0.692	0.408
L-DLPFC-L-FPC	0.001	0.982	0.197	0.658	0.248	0.620

Values represent F statistics and corresponding P-values from two-way ANOVA (Group × Lesion Side). ROI-pair abbreviations: R-FPC, right frontopolar cortex; L-FPC, left frontopolar cortex; R-DLPFC, right dorsolateral prefrontal cortex; L-DLPFC, left dorsolateral prefrontal cortex.

### 3.4 Hemodynamic responses during the VFT in PSA patients with and without comorbid depression

**Figure 5:**
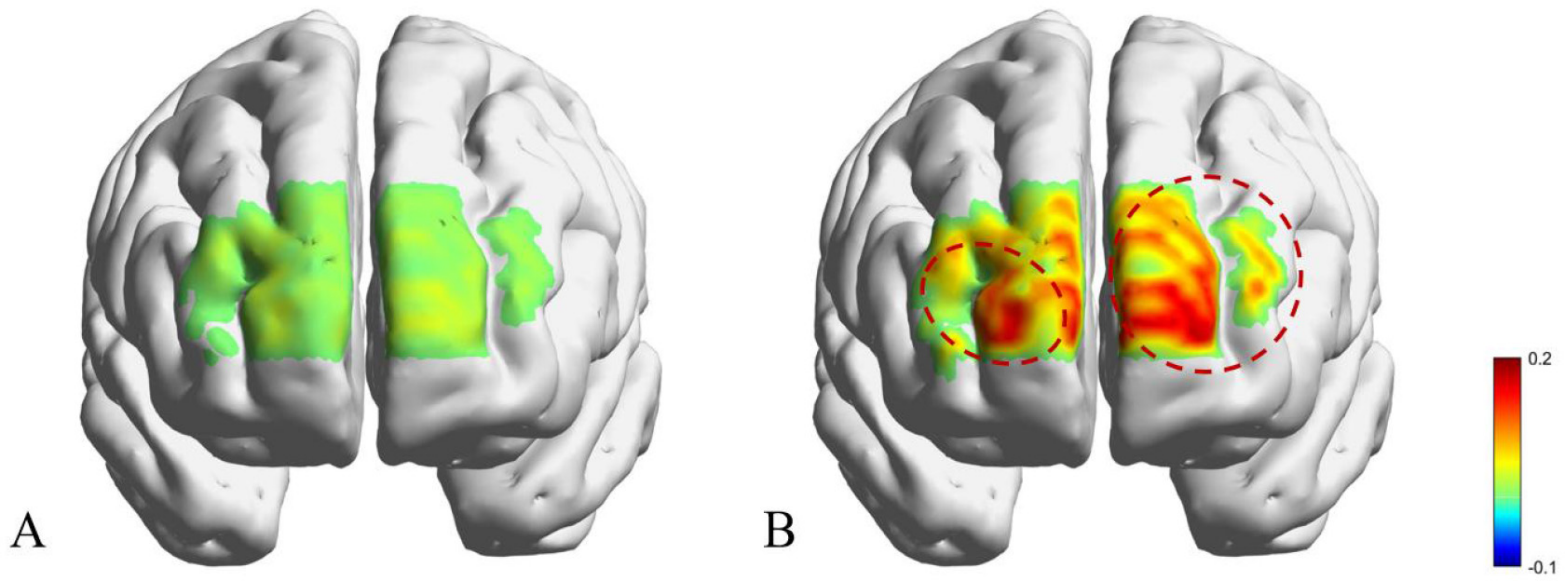
fNIRS-derived cortical activation maps of anxiety with depression and anxiety without depression groups during the VFT. **(A)** Anxiety with Depression Group; **(B)** Anxiety without Depression Group.

Group and lesion side effects on VFT-related prefrontal activation in PSA patients with and without comorbid depression (Tables 6, 7) were examined using two-way ANOVA. As shown in Tables 6, 7 and Figure 5, PSA patients with comorbid depression exhibited significantly lower activation in the bilateral FPC and left DLPFC compared with those without depression (*P* < 0.05), whereas no significant main effects of lesion side or group × lesion side interaction effects were observed (all *P* > 0.05).

**Table 6:** Brain activation in the VFT with and without depression in the anxiety groups.

**Variables**	**Anxiety with depression group**		**Anxiety without depression group**	
	**Left lesion (*****N*** = **11)**	**Right lesion (*****N*** = **23)**	**Left lesion (*****N*** = **8)**	**Right lesion (*****N*** = **8)**
R-FPC	−0.004 ± 0.168	0.031 ± 0.112	0.111 ± 0.177	0.154 ± 0.196
R-DLPFC	0.041 ± 0.109	0.016 ± 0.083	0.065 ± 0.119	0.089 ± 0.109
L-DLPFC	0.027 ± 0.102	0.013 ± 0.090	0.127 ± 0.190	0.074 ± 0.171
L-FPC	0.037 ± 0.136	0.030 ± 0.101	0.125 ± 0.220	0.171 ± 0.256

Values are presented as mean ± SD. R-FPC, right frontopolar cortex; L-FPC, left frontopolar cortex; R-DLPFC, right dorsolateral prefrontal cortex; L-DLPFC, left dorsolateral prefrontal cortex. Left lesion and right lesion refer to the hemispheric side of the stroke lesion.

**Table 7:** Statistical analysis of brain activation in the VFT with and without depression.

**Variables**	**Group effect**		**Lesion side effect**		**Interaction effect**	
	***F***	***P***	***F***	***P***	***F***	***P***
R-FPC	6.529	0.014	0.696	0.408	0.009	0.926
R-DLPFC	2.450	0.124	0.000	1.000	0.648	0.425
L-DLPFC	4.176	0.047	0.715	0.402	0.250	0.619
L-FPC	5.179	0.028	0.147	0.703	0.278	0.600

Values represent F statistics and corresponding P-values from two-way ANOVA (Group × Lesion Side). R-FPC, right frontopolar cortex; L-FPC, left frontopolar cortex; R-DLPFC, right dorsolateral prefrontal cortex; L-DLPFC, left dorsolateral prefrontal cortex.

### 3.5 Resting-state functional connectivity in PSA patients with and without comorbid depression

**Figure 6:**
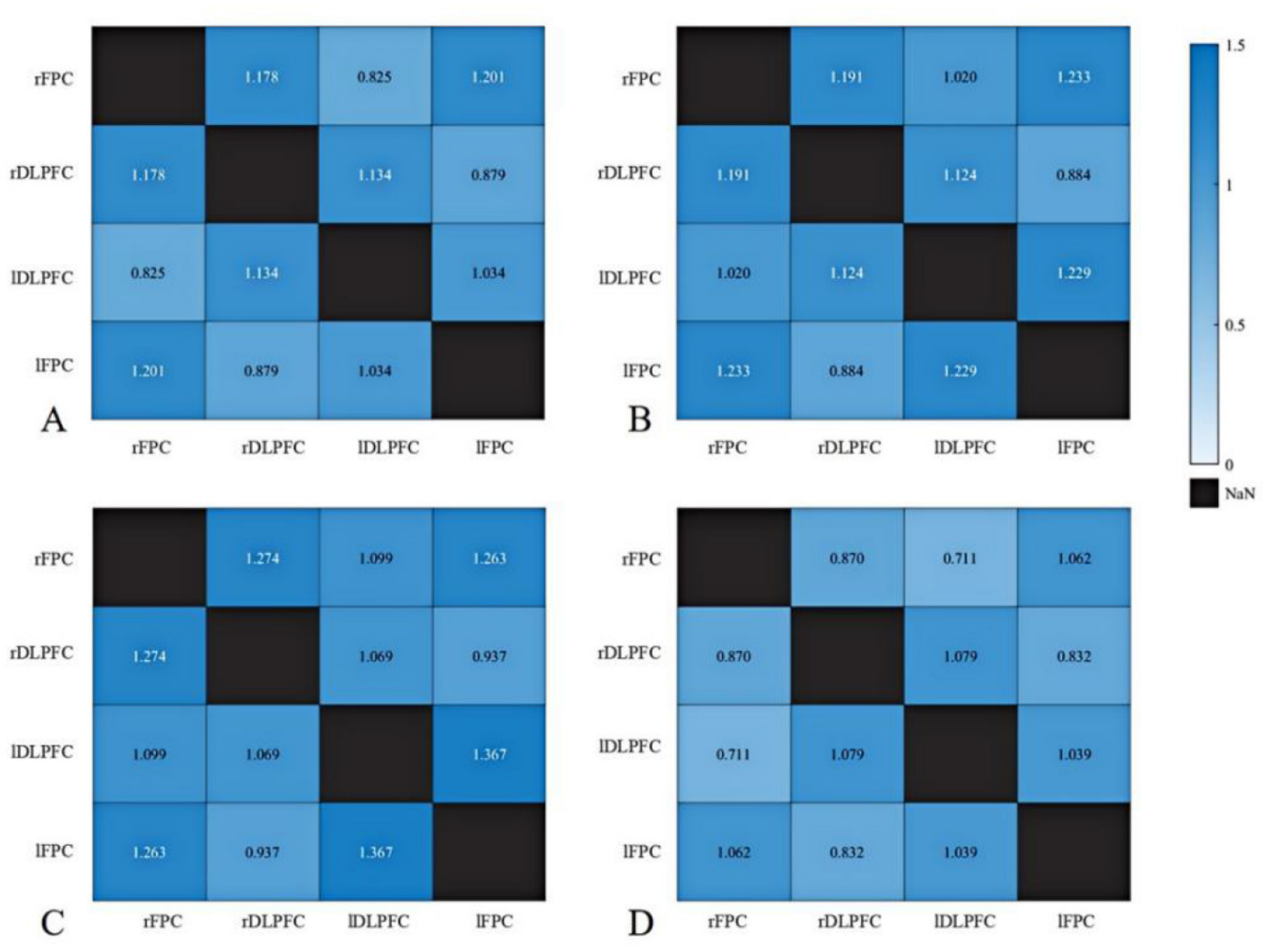
Resting-state brain functional connectivity (z-values) in individuals with and without depression. **(A)** Anxiety with depression group with left hemisphere injury; **(B)** Anxiety with depression group with right hemisphere injury; **(C)** Anxiety without depression group with left hemisphere injury; **(D)** Anxiety without depression group with right hemisphere injury.

Group and lesion side effects on resting-state prefrontal functional connectivity in PSA patients with and without comorbid depression (Table 8) were examined using two-way ANOVA. As shown in Table 8 and Figure 6, no significant main effects of group, lesion side, or group × lesion side interaction effects were observed (all *P* > 0.05).

**Table 8:** Statistical comparison of brain functional connectivity in the resting state with and without depression.

**Variables**	**Group effect**		**Lesion side effect**		**Interaction effect**	
	***F***	***P***	***F***	***P***	***F***	***P***
R-FPC-R-DLPFC	0.448	0.507	1.360	0.250	1.542	0.221
R-FPC-L-DLPFC	0.013	0.910	0.364	0.549	3.336	0.074
R-FPC-L-FPC	0.160	0.691	0.379	0.541	0.717	0.401
R-DLPFC -L-DLPFC	0.092	0.763	0.000	0.999	0.003	0.955
R-DLPFC-L-FPC	0.000	0.987	0.110	0.741	0.133	0.717
L-DLPFC-L-FPC	0.242	0.625	0.209	0.650	3.244	0.078

Values represent F statistics and corresponding P-values from two-way ANOVA (Group × Lesion Side). ROI-pair abbreviations: R-FPC, right frontopolar cortex; L-FPC, left frontopolar cortex; R-DLPFC, right dorsolateral prefrontal cortex; L-DLPFC, left dorsolateral prefrontal cortex.

## 4 Discussion

This study investigated the brain network characteristics of PSA patients using fNIRS. Furthermore, subgroup analyses revealed distinct activation patterns in brain regions during the VFT between PSA patients with and without comorbid depression, offering new insights into the potential neural mechanisms underlying PSA.

### 4.1 Comparison of brain activation between PSA and non-PSA patients

We found that PSA patients exhibited significantly lower bilateral FPC activation during the VFT compared to the non-PSA group. However, no significant differences in bilateral DLPFC activation were identified between the two groups. A two-way ANOVA further indicated no significant interaction effects, suggesting that the influence of anxiety on PFC activation is not modulated by lesion laterality in stroke patients.

The FPC, located at the most anterior part of the PFC, is among the most evolutionarily advanced brain regions and is involved in memory retrieval, relational reasoning, and executive decision-making (Wu et al., 2024). This study employed the VFT task, where patients are required to generate the maximum number of non-repetitive words based on semantic categories. This process depends on the integration of multidimensional cognitive resources regulated by the PFC (Kumar et al., 2017; Backes et al., 2014). The markedly reduced bilateral FPC activation observed in PSA patients suggests that this region may be a critical neural substrate underlying PSA. This result aligns with previous findings linking hemodynamic responses in the FPC and DLPFC to anxiety (Takizawa et al., 2014).

As a central hub connecting emotional and cognitive processes, the FPC has been shown to exhibit both structural and functional abnormalities in individuals with mood disorders (Hayashi et al., 2011; Takei et al., 2014). Our findings suggest two potential mechanisms through which anxiety may disrupt FPC function. First, from the perspective of cognitive control, increasing task demands may exacerbate anxiety-induced impairments in FPC-mediated executive control and processing efficiency, leading to reduced engagement of the FPC in task execution (Koechlin and Hyafil, 2007). Second, from a resource allocation perspective, anxiety may divert cognitive resources away from task performance and toward emotional regulation, particularly during high-demand tasks such as the VFT (Niendam et al., 2012; Zhao et al., 2024). This reallocation of resources could contribute to the diminished hemodynamic response observed in the FPC. Collectively, these mechanisms provide a neural basis for reduced FPC activation in PSA and offer theoretical support for developing targeted neuromodulatory interventions.

Although the DLPFC is essential for cognitive control, particularly in task switching and cognitive flexibility, no significant group differences in DLPFC activation were found in our study. This finding contrasts with previous studies on anxiety alone, which have reported negative correlations between anxiety severity and right DLPFC activation during the VFT (Zhao et al., 2024). A plausible explanation lies in the post-stroke population examined in our study. Stroke often induces both local and remote neural reorganization, which can reshape cognitive and affective processing networks (Tay et al., 2021; Qi et al., 2024). Consequently, PSA patients may compensate for impaired DLPFC function through functional recruitment of other related brain regions. Such neuroplastic remodeling may explain the absence of significant DLPFC activation differences observed in this study.

### 4.2 Comparison of brain activation between PSA patients with and without depression

Anxiety is often comorbid with depression, and both are frequently studied together in clinical and neuroimaging research (Wu et al., 2022). Post-stroke patients with depression often show overlapping anxiety symptoms, a phenomenon known as anxious depression (Wright et al., 2017). Given this overlap, it's crucial to examine whether comorbid depression alters the neural activation patterns in PSA.

Subgroup analysis revealed notable differences in brain activation during the VFT between PSA patients with and without depression. Specifically, those with comorbid depression showed significantly reduced activation in the bilateral FPC and L-DLPFC.

The DLPFC is a key region involved in emotional regulation, with both hemispheres contributing to this process. However, their functional roles differ depending on the stimulus: the L-DLPFC is more responsive to verbal stimuli, while the R-DLPFC is more attuned to non-verbal stimuli (White et al., 2023). These lateralized responses influence the activation patterns observed with fNIRS during the VFT. Previous research has demonstrated an inverse correlation between FPC activation and the severity of depression, with bilateral FPC deactivation in diagnosed depression and right-sided deactivation in subthreshold cases (Wu et al., 2024), consistent with our findings. Moreover, reduced L-DLPFC activation has been suggested as a potential marker for distinguishing PSD from non-PSD patients (Peng et al., 2023), which further supports our results.

However, other studies have identified R-DLPFC activation during the VFT as a potential neurophysiological marker of anxiety-related depression (Wu et al., 2022). This inconsistency could stem from methodological variations, especially the failure to account for the use of antidepressants. Pharmacological agents, including antidepressants, can influence the reliability and accuracy of fNIRS measurements (Takamiya et al., 2017). In our study, we addressed this potential confounding factor by rigorously excluding participants on psychotropic medications, thereby enhancing the interpretability of our neural findings.

### 4.3 Brain functional connectivity at rest

During the resting state, no significant differences in brain functional connectivity were found between PSA and non-PSA patients, nor between PSA patients with and without depression. These findings align with those from graph theory-based fNIRS studies (Peng et al., 2023), suggesting that neural alterations linked to emotional disorders are more readily detectable during cognitive or affective task engagement. This insight supports the development of standardized diagnostic protocols and the identification of therapeutic targets.

Nevertheless, the lack of group-level differences at rest does not suggest that PSA is free from connectivity abnormalities. Unlike primary anxiety or depression, stroke causes widespread alterations across the brain's structural and functional networks. Recovery involves complex processes of reorganization, remodeling, and metabolic compensation (Tay et al., 2021; Guggisberg et al., 2019). These dynamics may lead to diffuse rather than focal patterns of network reconfiguration, reflecting the brain's adaptive responses to structural damage and affective dysregulation. Therefore, a single neuroimaging modality may not fully capture the complexity of PSA-related mechanisms. Future studies may benefit from integrating multimodal approaches, such as fMRI or EEG, to enhance both spatial and temporal resolution and offer a deeper insight into the understanding of the altered brain networks underlying PSA.

## 5 Advantages and limitations

This study focused on prefrontal subregions in PSA patients, using fNIRS to examine cortical network characteristics during both task and resting states. It further compared PSA patients with and without depression, providing neurobiological evidence for differentiating post-stroke anxiety subtypes. These findings contribute to a better understanding of the relationship between PSA and depression.

From a clinical perspective, the identification of altered activation in the bilateral FPC in patients with PSA may provide a neurobiological basis for the development of future intervention strategies. Notably, additional involvement of the left DLPFC was observed specifically in PSA patients with comorbid depression, suggesting that distinct prefrontal subregions may be differentially associated with anxiety alone vs. anxiety accompanied by depressive symptoms. These findings indicate that modulation of prefrontal circuits, could be explored as complementary targets in future neuromodulation-based approaches. Future studies combining fNIRS with neuromodulation techniques, such as transcranial magnetic stimulation or transcranial direct current stimulation, are warranted to further investigate their therapeutic potential and clinical feasibility.

Several limitations should be noted. First, due to the technical constraints of fNIRS, this study was limited to cortical measurements and was unable to assess whole-brain networks. Specifically, fNIRS is inherently restricted in its ability to capture subcortical structures, which may also play an important role in post-stroke emotional regulation. Second, the number of word outputs in the VFT was not recorded, preventing evaluation of task engagement and limiting the interpretation of whether the observed neural differences were driven by anxiety-related mechanisms or variations in task performance; this represents an important methodological limitation.

Third, the exploratory and observational design, similar to that of Koyanagi et al. (2021), and the inclusion of patients with different stroke types may have potentially influenced the results. Stroke type represents a complex clinical characteristic that is closely related to lesion location, lesion extent, and overall disease severity. In the present sample, stroke type was unevenly distributed between groups, with relatively small numbers in certain subcategories, which limited the feasibility of statistically controlling for this factor without introducing model instability or over-adjustment. Although this reflects real-world clinical heterogeneity, the potential confounding effect of stroke type cannot be fully excluded, and the findings should therefore be interpreted with appropriate caution.

In addition, the subgroup analysis comparing PSA patients with and without comorbid depression involved very small sample sizes (as low as n = 8), which substantially limits statistical power and reduces the robustness of the subgroup findings. Moreover, although the final sample size was comparable to that of similar fNIRS studies, an a priori power calculation could not be performed due to the absence of reliable effect-size estimates specific to PSA, which may further limit the generalizability of the findings.

Future research should apply stricter inclusion criteria, incorporate behavioral performance measures, expand sample sizes, and conduct stratified or longitudinal analyses to more comprehensively clarify PSA-related neural mechanisms.

## 6 Conclusion

Bilateral FPC shows potential as a central target for evaluating and intervening in PSA, offering new directions for future treatment strategies. Moreover, functional abnormalities in both bilateral FPC and L-DLPFC could serve as potential biological markers for predicting PSA with depression.

This study suggests that altered activation of the bilateral frontopolar cortex (FPC) may represent an important neural substrate associated with post-stroke anxiety. In addition, functional alterations involving the bilateral FPC and left dorsolateral prefrontal cortex (L-DLPFC) were observed in PSA patients with comorbid depression, indicating their potential relevance to the neural correlates underlying this clinical subtype. Together, these findings provide preliminary neurobiological evidence that may inform future research on the assessment and stratification of PSA, as well as the development of targeted intervention strategies.

## Data Availability

The raw data supporting the conclusions of this article will be made available by the authors, without undue reservation.
